# A dataset for measuring the impact of research data and their curation

**DOI:** 10.1038/s41597-024-03303-2

**Published:** 2024-05-03

**Authors:** Libby Hemphill, Andrea Thomer, Sara Lafia, Lizhou Fan, David Bleckley, Elizabeth Moss

**Affiliations:** 1 Inter-university Consortium for Political and Social Research, University of Michigan, Ann Arbor, MI 48104 USA; 2 School of Information, University of Michigan, Ann Arbor, MI 48104 USA; 3 School of Information, University of Arizona, Tucson, AZ 85721 USA

**Keywords:** Social sciences, Research data

## Abstract

Science funders, publishers, and data archives make decisions about how to responsibly allocate resources to maximize the reuse potential of research data. This paper introduces a dataset developed to measure the impact of archival and data curation decisions on data reuse. The dataset describes 10,605 social science research datasets, their curation histories, and reuse contexts in 94,755 publications that cover 59 years from 1963 to 2022. The dataset was constructed from study-level metadata, citing publications, and curation records available through the Inter-university Consortium for Political and Social Research (ICPSR) at the University of Michigan. The dataset includes information about study-level attributes (e.g., PIs, funders, subject terms); usage statistics (e.g., downloads, citations); archiving decisions (e.g., curation activities, data transformations); and bibliometric attributes (e.g., journals, authors) for citing publications. This dataset provides information on factors that contribute to long-term data reuse, which can inform the design of effective evidence-based recommendations to support high-impact research data curation decisions.

## Background & Summary

Recent policy changes in funding agencies and academic journals have increased data sharing among researchers and between researchers and the public. Data sharing advances science and provides the transparency necessary for evaluating, replicating, and verifying results. However, many data-sharing policies do not explain what constitutes an appropriate dataset for archiving or how to determine the value of datasets to secondary users^1–3^. Questions about how to allocate data-sharing resources efficiently and responsibly have gone unanswered^4–6^. For instance, data-sharing policies recognize that not all data should be curated and preserved, but they do not articulate metrics or guidelines for determining what data are most worthy of investment.

Despite the potential for innovation and advancement that data sharing holds, the best strategies to prioritize datasets for preparation and archiving are often unclear. Some datasets are likely to have more downstream potential than others, and data curation policies and workflows should prioritize high-value data instead of being one-size-fits-all. Though prior research in library and information science has shown that the “analytic potential” of a dataset is key to its reuse value^7^, work is needed to implement conceptual data reuse frameworks^8–14^. In addition, publishers and data archives need guidance to develop metrics and evaluation strategies to assess the impact of datasets.

Several existing resources have been compiled to study the relationship between the reuse of scholarly products, such as datasets (Table 1); however, none of these resources include explicit information on how curation processes are applied to data to increase their value, maximize their accessibility, and ensure their long-term preservation. The CCex (Curation Costs Exchange) provides models of curation services along with cost-related datasets shared by contributors but does not make explicit connections between them or include reuse information^15^. Analyses on platforms such as DataCite^16^ have focused on metadata completeness and record usage, but have not included related curation-level information. Analyses of GenBank^17^ and FigShare^18,19^ citation networks do not include curation information. Related studies of Github repository reuse^20^ and Softcite software citation^21^ reveal significant factors that impact the reuse of secondary research products but do not focus on research data. RD-Switchboard^22^ and DSKG^23^ are scholarly knowledge graphs linking research data to articles, patents, and grants, but largely omit social science research data and do not include curation-level factors. To our knowledge, other studies of curation work in organizations similar to ICPSR – such as GESIS^24^, Dataverse^25^, and DANS^26^ – have not made their underlying data available for analysis.

**Table 1:** Comparison of existing datasets describing reuse and curation with ours (MICA).

Dataset	Disciplines	Description	Size	Includes curation info?
MICA (ours)	Social Science	Citation network; w/usage and curation stats	10 K datasets; 95 K papers	Yes - 669 data curation work logs
CCEx^15^	Digital curation	Platform for comparing curation costs	40 cost datasets	Yes - models of curation services
DataCite^16^	General	Metadata, data usage statistics	1.9 M datasets	No
GenBank^17^	Biology	Citation network	227 M annotations; 44.4 M papers; 42.5 patents	No
FigShare^18,19^	General	Citation network	18 K datasets; 7 K papers	No
Github^20^	Data Science	Github repos, files	1.5 M datasets; 65 K repositories	No
Softcite^21^	Biomedicine; Economics	Annotated paper corpus	4.9 K papers; 2.5 K citations	No
RD-Switchboard^22^	General	Citation network	144 K datasets; 2.8 M papers; 1 M researchers; 55 K grants	No
DSKG^23^	Computer Science; Biology	Citation network w/author info	2,000 datasets; 635 K papers	No

This paper describes a dataset^27^ compiled for the MICA project (Measuring the Impact of Curation Actions) led by investigators at ICPSR, a large social science data archive at the University of Michigan. The dataset was originally developed to study the impacts of data curation and archiving on data reuse. The MICA dataset has supported several previous publications investigating the intensity of data curation actions^28^, the relationship between data curation actions and data reuse^29^, and the structures of research communities in a data citation network^30^. Collectively, these studies help explain the return on various types of curatorial investments. The dataset that we introduce in this paper, which we refer to as the MICA dataset, has the potential to address research questions in the areas of science (e.g., knowledge production), library and information science (e.g., scholarly communication), and data archiving (e.g., reproducible workflows).

## Methods

**Fig. 1:**
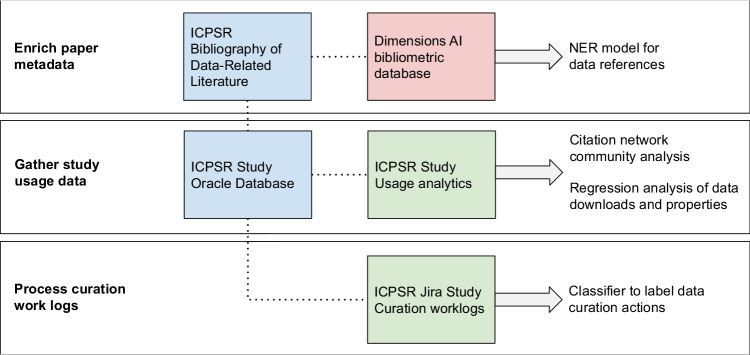
Steps to prepare MICA dataset for analysis - external sources are red, primary internal sources are blue, and internal linked sources are green.

We constructed the MICA dataset^27^ using records available at ICPSR, a large social science data archive at the University of Michigan. Data set creation involved: collecting and enriching metadata for articles indexed in the ICPSR Bibliography of Data-related Literature against the Dimensions AI bibliometric database; gathering usage statistics for studies from ICPSR’s administrative database; processing data curation work logs from ICPSR’s project tracking platform, Jira; and linking data in social science studies and series to citing analysis papers (Fig. 1).

### Enrich paper metadata

The ICPSR Bibliography of Data-related Literature is a growing database of literature in which data from ICPSR studies have been used. Its creation was funded by the National Science Foundation (Award 9977984), and for the past 20 years it has been supported by ICPSR membership and multiple US federally-funded and foundation-funded topical archives at ICPSR. The Bibliography was originally launched in the year 2000 to aid in data discovery by providing a searchable database linking publications to the study data used in them. The Bibliography collects the universe of output based on the data shared in each study through, which is made available through each ICPSR study’s webpage. The Bibliography contains both peer-reviewed and grey literature, which provides evidence for measuring the impact of research data. For an item to be included in the ICPSR Bibliography, it must contain an analysis of data archived by ICPSR or contain a discussion or critique of the data collection process, study design, or methodology^31^. The Bibliography is manually curated by a team of librarians and information specialists at ICPSR who enter and validate entries. Some publications are supplied to the Bibliography by data depositors, and some citations are submitted to the Bibliography by authors who abide by ICPSR’s terms of use requiring them to submit citations to works in which they analyzed data retrieved from ICPSR. Most of the Bibliography is populated by Bibliography team members, who create custom queries for ICPSR studies performed across numerous sources, including Google Scholar, ProQuest, SSRN, and others. Each record in the Bibliography is one publication that has used one or more ICPSR studies. The version we used was captured on 2021-11-16 and included 94,755 publications.

To expand the coverage of the ICPSR Bibliography, we searched exhaustively for all ICPSR study names, unique numbers assigned to ICPSR studies, and DOIs^32^ using a full-text index available through the Dimensions AI database^33^. We accessed Dimensions through a license agreement with the University of Michigan. ICPSR Bibliography librarians and information specialists manually reviewed and validated new entries that matched one or more search criteria. We then used Dimensions to gather enriched metadata and full-text links for items in the Bibliography with DOIs. We matched 43% of the items in the Bibliography to enriched Dimensions metadata including abstracts, field of research codes, concepts, and authors’ institutional information; we also obtained links to full text for 16% of Bibliography items. Based on licensing agreements, we included Dimensions identifiers and links to full text so that users with valid publisher and database access can construct an enriched publication dataset.

### Gather study usage data

ICPSR maintains a relational administrative database, DBInfo, that organizes study-level metadata and information on data reuse across separate tables. Studies at ICPSR consist of one or more files collected at a single time or for a single purpose; studies in which the same variables are observed over time are grouped into series. Each study at ICPSR is assigned a DOI, and its metadata are stored in DBInfo. Study metadata follows the Data Documentation Initiative (DDI) Codebook 2.5 standard. DDI elements included in our dataset are title, ICPSR study identification number, DOI, authoring entities, description (abstract), funding agencies, subject terms assigned to the study during curation, and geographic coverage. We also created variables based on DDI elements: total variable count, the presence of survey question text in the metadata, the number of author entities, and whether an author entity was an institution. We gathered metadata for ICPSR’s 10,605 unrestricted public-use studies available as of 2021-11-16 (https://www.icpsr.umich.edu/web/pages/membership/or/metadata/oai.html).

To link study usage data with study-level metadata records, we joined study metadata from DBinfo on study usage information, which included total study downloads (data and documentation), individual data file downloads, and cumulative citations from the ICPSR Bibliography. We also gathered descriptive metadata for each study and its variables, which allowed us to summarize and append recoded fields onto the study-level metadata such as curation level, number and type of principle investigators, total variable count, and binary variables indicating whether the study data were made available for online analysis, whether survey question text was made searchable online, and whether the study variables were indexed for search. These characteristics describe aspects of the discoverability of the data to compare with other characteristics of the study. We used the study and series numbers included in the ICPSR Bibliography as unique identifiers to link papers to metadata and analyze the community structure of dataset co-citations in the ICPSR Bibliography^32^.

### Process curation work logs

Researchers deposit data at ICPSR for curation and long-term preservation. Between 2016 and 2020, more than 3,000 research studies were deposited with ICPSR. Since 2017, ICPSR has organized curation work into a central unit that provides varied levels of curation that vary in the intensity and complexity of data enhancement that they provide. While the levels of curation are standardized as to effort (level one = less effort, level three = most effort), the specific curatorial actions undertaken for each dataset vary. The specific curation actions are captured in Jira, a work tracking program, which data curators at ICPSR use to collaborate and communicate their progress through tickets. We obtained access to a corpus of 669 completed Jira tickets corresponding to the curation of 566 unique studies between February 2017 and December 2019^28^.

To process the tickets, we focused only on their work log portions, which contained free text descriptions of work that data curators had performed on a deposited study, along with the curators’ identifiers, and timestamps. To protect the confidentiality of the data curators and the processing steps they performed, we collaborated with ICPSR’s curation unit to propose a classification scheme, which we used to train a Naive Bayes classifier and label curation actions in each work log sentence. The eight curation action labels we proposed^28^ were: (1) initial review and planning, (2) data transformation, (3) metadata, (4) documentation, (5) quality checks, (6) communication, (7) other, and (8) non-curation work. We note that these categories of curation work are very specific to the curatorial processes and types of data stored at ICPSR, and may not match the curation activities at other repositories. After applying the classifier to the work log sentences, we obtained summary-level curation actions for a subset of all ICPSR studies (5%), along with the total number of hours spent on data curation for each study, and the proportion of time associated with each action during curation.

## Data Records

**Fig. 2:**
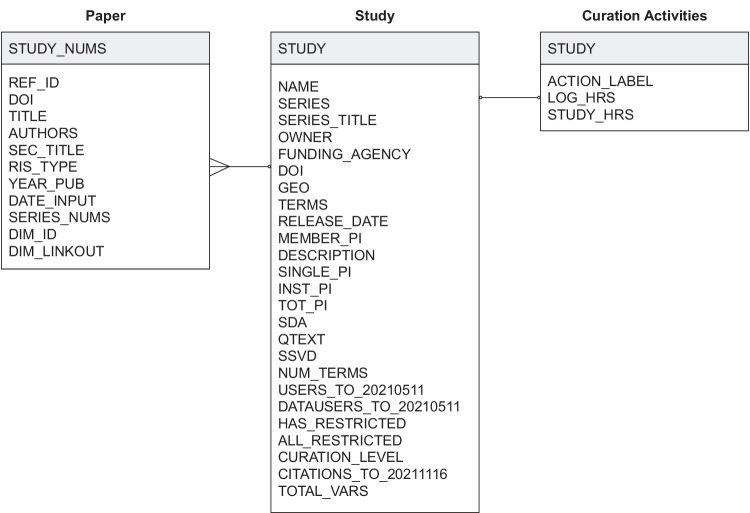
Entity-relation diagram.

The MICA dataset^27^ connects records for each of ICPSR’s archived research studies to the research publications that use them and related curation activities available for a subset of studies (Fig. 2). Each of the three tables published in the dataset is available as a study archived at ICPSR. The data tables are distributed as statistical files available for use in SAS, SPSS, Stata, and R as well as delimited and ASCII text files. The dataset is organized around studies and papers as primary entities. The studies table lists ICPSR studies, their metadata attributes, and usage information; the papers table was constructed using the ICPSR Bibliography and Dimensions database; and the curation logs table summarizes the data curation steps performed on a subset of ICPSR studies.

- **Studies** (“ICPSR_STUDIES”): 10,605 social science research datasets available through ICPSR up to 2021-11-16 with variables for ICPSR study number, digital object identifier, study name, series number, series title, authoring entities, full-text description, release date, funding agency, geographic coverage, subject terms, topical archive, curation level, single principal investigator (PI), institutional PI, the total number of PIs, total variables in data files, question text availability, study variable indexing, level of restriction, total unique users downloading study data files and codebooks, total unique users downloading data only, and total unique papers citing data through November 2021. Studies map to the papers and curation logs table through ICPSR study numbers as “STUDY”. However, not every study in this table will have records in the papers and curation logs tables.
- **Papers** (“ICPSR_PAPERS”): 94,755 publications collected from 2000-08-11 to 2021-11-16 in the ICPSR Bibliography and enriched with metadata from the Dimensions database with variables for paper number, identifier, title, authors, publication venue, item type, publication date, input date, ICPSR series numbers used in the paper, ICPSR study numbers used in the paper, the Dimension identifier, and the Dimensions link to the publication’s full text. Papers map to the studies table through ICPSR study numbers in the “STUDY_NUMS” field. Each record represents a single publication, and because a researcher can use multiple datasets when creating a publication, each record may list multiple studies or series.
- **Curation logs** (“ICPSR_CURATION_LOGS”): 649 curation logs for 563 ICPSR studies (although most studies in the subset had one curation log, some studies were associated with multiple logs, with a maximum of 10) curated between February 2017 and December 2019 with variables for study number, action labels assigned to work description sentences using a classifier trained on ICPSR curation logs, hours of work associated with a single log entry, and total hours of work logged for the curation ticket. Curation logs map to the study and paper tables through ICPSR study numbers as “STUDY”. Each record represents a single logged action, and future users may wish to aggregate actions to the study level before joining tables.

## Technical Validation

We report on the reliability of the dataset’s metadata in the following subsections. To support future reuse of the dataset, curation services provided through ICPSR improved data quality by checking for missing values, adding variable labels, and creating a codebook.

All 10,605 studies available through ICPSR have a DOI and a full-text description summarizing what the study is about, the purpose of the study, the main topics covered, and the questions the PIs attempted to answer when they conducted the study. Personal names (i.e., principal investigators) and organizational names (i.e., funding agencies) are standardized against an authority list maintained by ICPSR; geographic names and subject terms are also standardized and hierarchically indexed in the ICPSR Thesaurus^34^. Many of ICPSR’s studies (63%) are in a series and are distributed through the ICPSR General Archive (56%), a non-topical archive that accepts any social or behavioral science data. While study data have been available through ICPSR since 1962, the earliest digital release date recorded for a study was 1984-03-18, when ICPSR’s database was first employed, and the most recent date is 2021-10-28 when the dataset was collected.

**Fig. 3:**
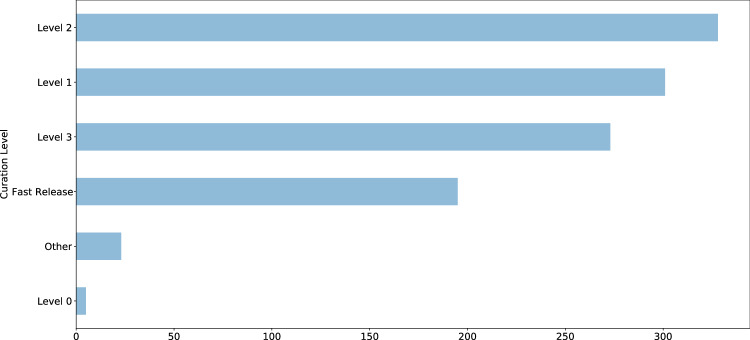
ICPSR study curation levels.

**Fig. 4:**
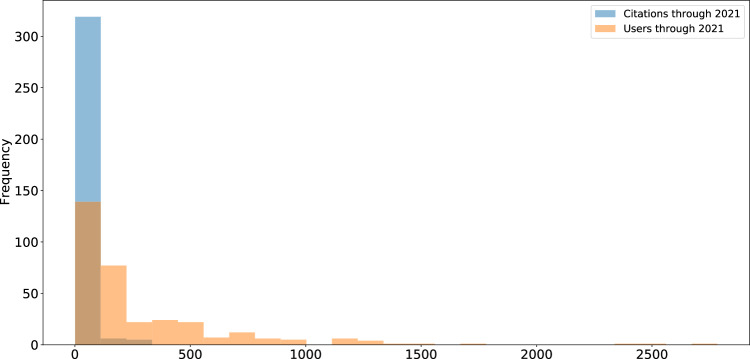
ICPSR study usage.

Curation level information was recorded starting in 2017 and is available for 1,125 studies (11%); approximately 80% of studies with assigned curation levels received curation services, equally distributed between Levels 1 (least intensive), 2 (moderately intensive), and 3 (most intensive) (Fig. 3). Detailed descriptions of ICPSR’s curation levels are available online^35^. Additional metadata are available for a subset of 421 studies (4%), including information about whether the study has a single PI, an institutional PI, the total number of PIs involved, total variables recorded is available for online analysis, has searchable question text, has variables that are indexed for search, contains one or more restricted files, and whether the study is completely restricted. We provided additional metadata for this subset of ICPSR studies because they were released within the past five years and detailed curation and usage information were available for them. Usage statistics including total downloads and data file downloads are available for this subset of studies as well; citation statistics are available for 8,030 studies (76%). Most ICPSR studies have fewer than 500 users, as indicated by total downloads, or citations (Fig. 4).

**Fig. 5:**
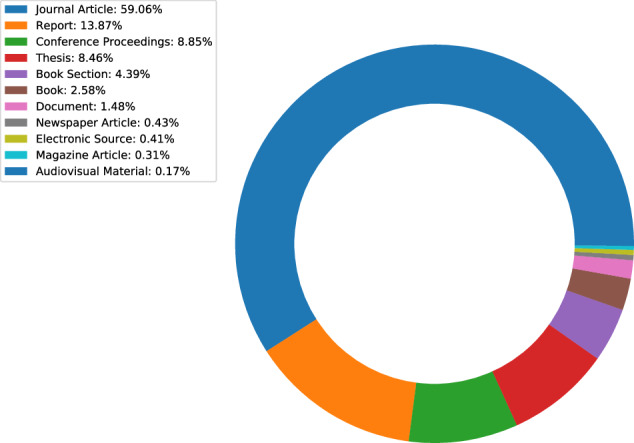
ICPSR Bibliography citation types.

**Fig. 6:**
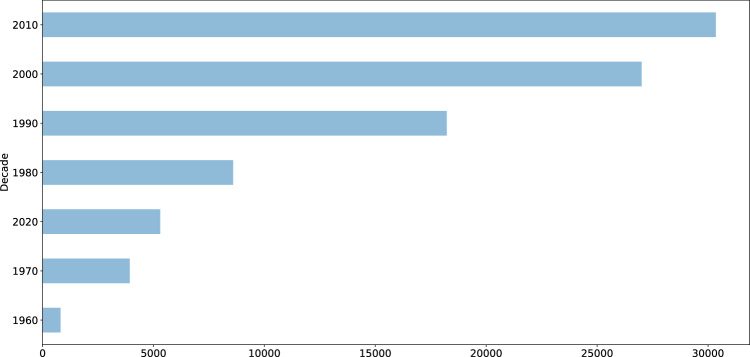
ICPSR citations by decade.

A subset of 43,102 publications (45%) available in the ICPSR Bibliography had a DOI. Author metadata were entered as free text, meaning that variations may exist and require additional normalization and pre-processing prior to analysis. While author information is standardized for each publication, individual names may appear in different sort orders (e.g., “Earls, Felton J.” and “Stephen W. Raudenbush”). Most of the items in the ICPSR Bibliography as of 2021-11-16 were journal articles (59%), reports (14%), conference presentations (9%), or theses (8%) (Fig. 5). The number of publications collected in the Bibliography has increased each decade since the inception of ICPSR in 1962 (Fig. 6). Most ICPSR studies (76%) have one or more citations in a publication.

## Usage Notes

The dataset consists of three tables that can be joined using the “STUDY” key as shown in Fig. 2. The “ICPSR_PAPERS” table contains one row per paper with one or more cited studies in the “STUDY_NUMS” column. We manipulated and analyzed the tables as CSV files with the Pandas library^36^ in Python and the Tidyverse packages^37^ in R.

The present MICA dataset can be used independently to study the relationship between curation decisions and data reuse. Evidence of reuse for specific studies is available in several forms: usage information, including downloads and citation counts; and citation contexts within papers that cite data. Analysis may also be performed on the citation network formed between datasets and papers that use them. Finally, curation actions can be associated with properties of studies and usage histories.

This dataset has several limitations of which users should be aware. First, Jira tickets can only be used to represent the intensiveness of curation for activities undertaken since 2017, when ICPSR started using both Curation Levels and Jira. Studies published before 2017 were all curated, but documentation of the extent of that curation was not standardized and therefore could not be included in these analyses. Second, the measure of publications relies upon the authors’ clarity of data citation and the ICPSR Bibliography staff’s ability to discover citations with varying formality and clarity. Thus, there is always a chance that some secondary-data-citing publications have been left out of the bibliography. Finally, there may be some cases in which a paper in the ICSPSR bibliography did not actually obtain data from ICPSR. For example, PIs have often written about or even distributed their data prior to their archival in ICSPR. Therefore, those publications would not have cited ICPSR but they are still collected in the Bibliography as being directly related to the data that were eventually deposited at ICPSR.

In summary, the MICA dataset contains relationships between two main types of entities – papers and studies – which can be mined. The tables in the MICA dataset have supported network analysis (community structure and clique detection)^30^; natural language processing (NER for dataset reference detection)^32^; visualizing citation networks (to search for datasets)^38^; and regression analysis (on curation decisions and data downloads)^29^. The data are currently being used to develop research metrics and recommendation systems for research data. Given that DOIs are provided for ICPSR studies and articles in the ICPSR Bibliography, the MICA dataset can also be used with other bibliometric databases, including DataCite, Crossref, OpenAlex, and related indexes. Subscription-based services, such as Dimensions AI, are also compatible with the MICA dataset. In some cases, these services provide abstracts or full text for papers from which data citation contexts can be extracted for semantic content analysis.

## Data Availability

The code^27^ used to produce the MICA project dataset is available on GitHub at https://github.com/ICPSR/mica-data-descriptor and through Zenodo with the identifier 10.5281/zenodo.8432666. Data manipulation and pre-processing were performed in Python. Data curation for distribution was performed in SPSS.
